# Evaluation of commercial and automated SARS-CoV-2 IgG and IgA ELISAs using coronavirus disease (COVID-19) patient samples

**DOI:** 10.2807/1560-7917.ES.2020.25.18.2000603

**Published:** 2020-05-07

**Authors:** Anne J Jääskeläinen, Eliisa Kekäläinen, Hannimari Kallio-Kokko, Laura Mannonen, Elisa Kortela, Olli Vapalahti, Satu Kurkela, Maija Lappalainen

**Affiliations:** 1 Department of Virology, University of Helsinki and Helsinki University Hospital, Helsinki, Finland; 2 Infectious diseases, University of Helsinki and Helsinki University Hospital, Helsinki, Finland; 3 These authors contributed equally to the work

**Keywords:** SARS-CoV-2, COVID-19, serology, commercial, IgG, IgA

## Abstract

Antibody-screening methods to detect severe acute respiratory syndrome coronavirus 2 (SARS-CoV-2) need to be validated. We evaluated SARS-CoV-2 IgG and IgA ELISAs in conjunction with the EUROLabworkstation (Euroimmun, Lübeck, Germany). Overall specificities were 91.9% and 73.0% for IgG and IgA ELISAs, respectively. Of 39 coronavirus disease patients, 13 were IgG and IgA positive and 11 IgA alone at sampling. IgGs and IgAs were respectively detected at a median of 12 and 11 days after symptom onset.

Severe acute respiratory syndrome coronavirus 2 (SARS-CoV-2) causes coronavirus disease (COVID-19) [1]. At time of writing on 22 April 2020, the COVID-19 pandemic had resulted in approximatively 2.5 million reported cases worldwide with over 177,000 deaths [1].

Fast and reliable laboratory diagnostics for SARS-CoV2 [2-4] are important to support the rapid implementation of appropriate public health interventions. In the acute phase of COVID-19, laboratory diagnostics primarily rely on molecular methods [5,6]. In addition, serological assays are now being developed to allow epidemiological assessments through serosurveys, as well as retrospective diagnosis in targeted groups. The need for high quality testing kits suitable for in vitro diagnostics (IVD), automated laboratory equipment and laboratory information systems (LIS) is urgent. LIS, which record, manage, and store data are one of the key elements in reliable diagnostics with high throughput. Here we report on the evaluation of an automated serological screening approach for SARS-CoV-2 IgG and IgA antibodies.

## Sample collection for evaluation of SARS-CoV-2 IgG and IgA assays

We evaluated two commercial kits designed to respectively detect SARS-CoV-2 IgG and IgA antibodies in patient samples (CE marked in vitro diagnostic products; SARS-CoV-2 IgG and IgA ELISAs, Euroimmun, Lübeck, Germany; www.euroimmun.com). These commercial immunoassays are based on recombinant structural protein (S1) from SARS-CoV-2 and the S1-based ELISAs have been set up and tested for cross-reactions against other HCoVs [7,8]. In the current study, the kits were used in combination with the automated EUROLabworkstation (Euroimmun) for ELISA analysis with LIS.

To estimate specificity, we retrospectively used a panel of 37 patient sera from 15 male and 22 female patients (median age: 53 years; range: 5-87) collected in 2019 and 2020, which were considered negative for SARS-CoV-2. Among these, 11 serum samples were from patients who had been diagnosed with seasonal human coronaviruses (HCoVs: OC43, NL63, 229E) or other respiratory viruses by nucleic acid tests (NAT). Four of these 11 samples, which originated from patients testing positive for HCoV, had been collected in 2019. The rest were from 2020. The four samples from 2019 were assumed to be from SARS-CoV-2 negative patients, while all the samples obtained in 2020 were from patients who had been tested for SARS-CoV-2 nucleic acid and found negative. The remaining 26 of the 37 serum samples originated from patients who had been diagnosed as having adenovirus, enterovirus, influenza A, influenza B, parainfluenza, or respiratory syncytial (RSV) virus infections, through routine IgG antibody testing in 2019. These samples were assumed to be negative for SARS-CoV-2. 

To investigate the output of the immunoassays on samples from individuals who had been infected with SARS-CoV-2, we retrospectively collected serum samples from patients, who had been prior diagnosed with COVID-19 by real-time RT-PCRs (RT-qPCR) on nasopharyngeal samples as described by Corman et al. [6]. In total, 47 serum samples from 40 individuals (23 males, 17 females) were included. The median age of the patients was 56 years (range: 24–77 years). 

For 37 of these 40 individuals, a list of symptoms was available, enabling to rate their disease severity (Table 1). The demographic characteristics of these patients are shown in Table 1 and their samples were employed to study serological results according to disease severity [9].

**Table 1:** Demographic data of COVID-19 patients considered in the study and severity of disease Finland, 2020 (n = 40 patients)

Disease severity^a^ (proportion of patients)	ID	Sex	Proportion of M and F	Age in years	Median age in years (range)
Mild (9/37^b^)	1	M	M: 3/9 F: 6/9	68	41 (24–68)
	2	F		32	
	4	F		32	
	5	F		24	
	6	F		50	
	12	F		51	
	20	F		24	
	21	M		59	
	28	M		41	
Moderate (15/37^b^)	3	M	M: 8/15 F: 7/15	34	56 (30–79)
	7	M		77	
	8	M		53	
	10	M		59	
	13	F		56	
	15	M		54	
	17	M		75	
	22	F		49	
	23	F		50	
	26	F		79	
	30	F		67	
	33	F		30	
	35	M		65	
	36	F		34	
	38	M		60	
Severe (13/37^b^)	14	F	M: 10/13 F: 3/13	43	57 (39–72)
	16	M		72	
	19	M		64	
	24	M		50	
	25	M		39	
	27	M		50	
	29	M		71	
	31	M		58	
	32	F		57	
	34	M		66	
	37	F		56	
	39	M		66	
	40	M		45	
Not available (3/40)	9	F	M: 2/3 F: 1/3	33	64 (33–77)
	11	M		64	
	18	M		77	
**Total (n = 40 patients)**	**NA**	**NA**	**M: 23/40** **F: 17/40**	**NA**	**56 (24–77)**

COVID-19: coronavirus disease; F: female; ID: identity; M: male; NA: not applicable.

^a ^Symptom severity based on Siddiqi and Mehra (2020, in press) [9].

^b^ The denominator is based on 37 patients with information on disease severity.

Finally, we used immunoassays to investigate 13 sera of probable COVID-19 patients (according to the World Health Organization definition [10]) from February–March 2020, who had tested negative for SARS-CoV-2 by NAT.

Data were collected and samples handled according to research permit HUS/32/2018 (Helsinki University Hospital, Finland).

The specimens were analysed with SARS-CoV-2 IgG and IgA kits (Euroimmun) on the EUROLabworkstation (Euroimmun) platform. 

## Specificity of SARS-COV-2 IgG and IgA assays

From the panel of 37 sera considered negative for SARS-CoV-2, serologically negative results for IgG were found in 34 samples and for IgA in 27 samples, yielding a specificity of 91.9% for IgG and 73.0% for IgA (Table 2). Serum samples from one patient with HCoV OC43 infection showed a cross-reaction, however, cross-reactions were not observed in patients with HCoV 229E or NL63 infection. 

**Table 2:** Results of Euroimmun SARS-CoV-2 IgG and IgA ELISAs on patient sera collected in 2019–2020 and specificity of these assays, Finland, 2020 (n = 37)

Description of samples positive for another virus than SARS-CoV-2			SARS-CoV-2 Euroimmun ratio^a^ result (median ratio; range)		Specificity of Euroimmun tests	
Method (No individuals)	Virus (year)	No samples positive for the virus	IgG	IgA	IgG	IgA
IgG (n = 26 individuals)	Influenza A virus (2019)	26	**24 negative** (0.25; 0.13–0.76) **2 inconclusive** (1.02 0.96–1.07) **0 positive** (0; NA)	**19 negative** ****(0.31; 0.09–0.77) **2 inconclusive** (1.05; 1.02–1.07) **5 positive** (5.12; 1.52–7.96)	**24/26**	**19/26**
	Influenza B virus (2019)	26				
	Parainfluenza virus (2019)	26				
	RSV (2019)	26				
	Enterovirus (2019)	25				
	Adenovirus (2019)	24				
NAT (n = 11 individuals)	RSV (2020)^b^	1	**1 negative** (0.36; NA)	**1 positive** (2.69; NA)	**10/11**	**8/11**
	RSV and human bocavirus (2020)^b^	1	**1 negative** (0.27; NA)	**1 negative** (0.31; NA)		
	Adenovirus (2020)^b^	1	**1 negative** (0.20; NA)	**1 negative** (0.17; NA)		
	Adeno- and rhinovirus (2020)^b^	1	**1 negative** (0.45; NA)	**1 negative** (0.35; NA)		
	Human coronavirus OC43^c^ (2019; 2020^b^)	5	**4 negative** (0.20; 0.13-0.25) **0 inconclusive** (NA; NA) **1 positive** (2.54; NA)^d^	**3 negative** (0.12; 0.09-0.25) **1 inconclusive** (0.97; NA) **1 positive** (1.22; NA)^d^		
	Human coronavirus NL63 (2020)^b^	1	**1 negative** (0.32; NA)	**1 negative** (0.36; NA)		
	Human coronavirus 229E (2020)^b^	1	**1 negative** (0.22; NA)	**1 negative** (0.33; NA)		
**Overall specificity**					**34/37 (91.9%)**	**27/37 (73.0%)**

No: number; NAT: nucleic acid test; RSV: respiratory syncytial virus; SARS-CoV-2: severe acute respiratory syndrome coronavirus 2.

^a ^The ratios (between the extinction of the sample and calibrator) are the signals given by the assays. A ratio < 0.8 is considered negative, ≥ 0.8 and < 1.1 inconclusive and ≥ 1.1 positive.

^b^ The patient tested negative for SARS-CoV-2 nucleic acid.

^c^ Of the five samples of human coronavirus OC43, four were collected in 2019 and one was collected in 2020. The sample from 2020 was from a patient, who was tested for SARS-CoV-2 nucleic acid and found negative.

^d^ Same individual from year 2019.

Samples collected in 2019 were assumed to be from individuals who were not or had not been infected with SARS-CoV-2.

## Results from confirmed COVID-19 patients

**Figure 1:**
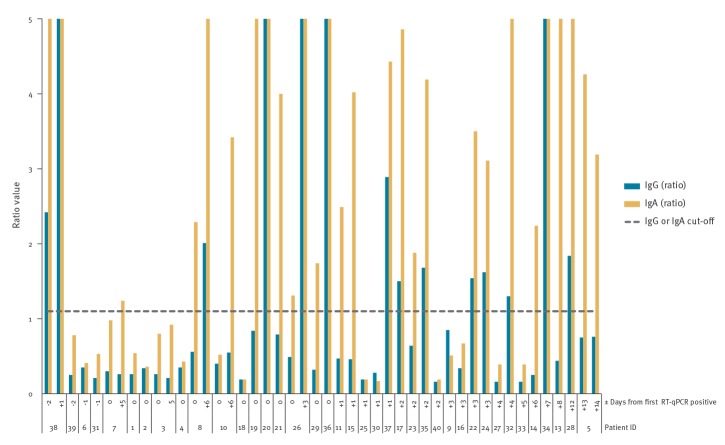
Distribution of IgG and IgA ratio values (Euroimmun) from COVID-19 patients according to time after first positive PCR test result, Finland, 2020 (n = 39 patients)

COVID-19: coronavirus disease; ID: patient identification; RT-qPCR: real-time RT-PCR.

A total of 40 patients, whose serum samples were included in the study, had been diagnosed with COVID-19 by RT-qPCR. One patient with a single sample taken before symptom onset was not further investigated. The IgG and IgA respective Euroimmun ratio values obtained from samples of the remaining 39 patients are shown relative to the time elapsed from the first positive PCR test result in Figure 1. It is notable, that for a patient with mild disease (ID 5) (Table 1), the serum sample was negative by IgG assay even 14 days after the first SARS-CoV-2 PCR-positive results (corresponding to 16 days after onset of symptoms); this sample was nevertheless positive for IgA (Figure 1).

The ratio values (between the extinction of the sample and calibrator) are the signals given by the assays. A ratio < 0.8 is considered negative, ≥ 0.8 and < 1.1 inconclusive and ≥ 1.1 positive. A dotted line indicates the 1.1 cut-off in the Figure.

The maximum IgG or IgA ratio value is 5 in this figure. Patient ID 12 is not included in the figure as the only serum sample was taken in January 2020, 66 days before onset of symptoms.

**Figure 2:**
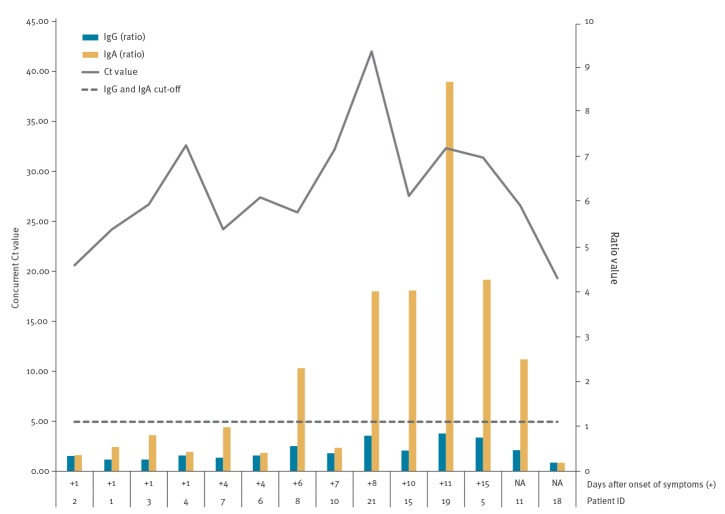
SARS-CoV-2 IgG and IgA ELISA ratio values (Euroimmun) and RT-qPCR Ct values from concurrent serum and nasopharyngeal samples, Finland, 2020 (n = 14)

For 14 COVID-19 patients, a serum sample happened to have been collected on the same date as the nasopharyngeal sample used for SARS-CoV-2 RT-qPCR: none of the 14 serum samples showed IgG antibodies, while six showed IgA antibodies (Figure 2). The nasopharyngeal and serum samples were taken at a median of 5 days (range: 1–15) after onset of symptoms. Generally, IgA positivity seemed to be detected earlier than that of IgG and samples appeared more frequently positive with IgA than IgG (Figure 1, Figure 2).

Ct: cycle threshold; NA: not available; RT-qPCR: real-time RT-PCR; SARS-CoV-2: severe acute respiratory syndrome coronavirus 2.

The ratio values (between the extinction of the sample and calibrator) are the signals given by the assays. A ratio < 0.8 is considered negative, ≥ 0.8 and < 1.1 inconclusive and ≥ 1.1 positive. A dotted line indicates the 1.1 cut-off in the figure.

For patient ID 21, the Ct value is above 40, but the RT-qPCR result was repeatedly positive and therefore the sample of this patient is considered to be PCR-positive.

**Figure 3:**
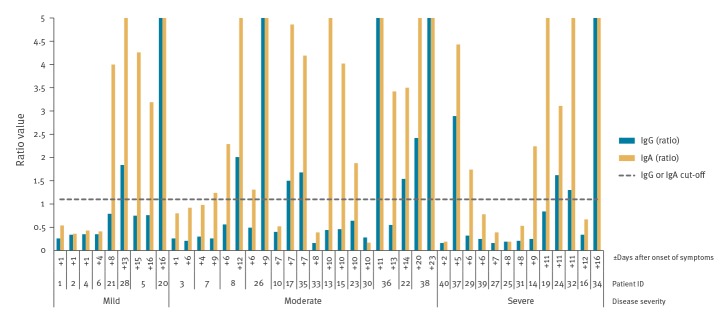
SARS-CoV-2 IgG and IgA ELISA ratio values (Euroimmun) from COVID-19 patients with data of disease severity and days after onset of symptoms, Finland, 2020 (n = 37)

Among the 39 COVID-19 patients screened for antibodies against SARS-CoV-2, IgAs and IgGs were both detected in 13, while IgAs alone were detected in 11, yielding a total of 24 IgA positive patients. The median time after onset of symptoms was 12 days (13 patients range: 5–20 days) for detection of IgGs, and 11 days (24 patients range: 5–20 days) for detection of IgAs (Figure 3).

ID: patient code.

The ratio values (between the extinction of the sample and calibrator) are the signals given by the assays. A ratio < 0.8 is considered negative, ≥ 0.8 and < 1.1 inconclusive and ≥ 1.1 positive. A dotted line indicates the 1.1 cut-off in the figure.

The maximum IgG and IgA ratio value is 5 in this figure. Disease severity data were available from 37 of 40 COVID-19 patients (listed in Table 1). Patient IDs 9, 11, and 18 not in this figure. In addition, patient ID 12 is not included in the figure as the only serum sample was taken in January 2020, 66 days before onset of symptoms.

A total of 37 patients had, in addition to the date of symptom onset, a list of symptoms available, allowing us to categorise the severity of their disease by COVID-19 as mild, moderate or severe [9] (Table 1, Figure 3). We did not see any clear patterns between the IgG or IgA results and disease severity, however the patient number was low (Figure 3).

## Results from probable COVID-19 patients

A number of individuals had returned from a COVID-19 epidemic area in Europe or China and had fever and other symptoms compatible with respiratory infection, leading them to be considered as probable COVID-19 patients, however their NAT on nasopharyngeal samples was negative for SARS-CoV-2 as well as for other respiratory viruses tested (Table 3). For 13 of these patients, concurrent nasopharyngeal and serum samples were available for serological testing: one patient was positive for both IgG and IgA in the serum sample, one patient for IgG alone, and one patient IgA alone (Table 3). No further samples from these patients were available for testing. 

**Table 3:** SARS-CoV-2 IgG and IgA ELISA ratio values (Euroimmun) on probable COVID-19 patients who had concurrent nasopharyngeal and serum samples taken but who tested SARS-CoV-2 RT-qPCR negative, Finland, February–March 2020 (n = 13)

**Respiratory virus for which the patient sample was NAT-negative **	**No of individuals**	**SARS-CoV-2 IgG result** **(Euroimmun median ratio^a^ ; range)**	**SARS-CoV-2 IgA result ** **(Euroimmun median ratio^a^ ; range)**
Influenza A virus	13	10 negative (0.27; 0.14–0.45) 1 inconclusive (0.87; NA) 2 positive (1.81; 1.58–2.04)	11 negative (0.32; 0.12–0.68) 0 inconclusive (NA; NA) 2 positive (2.90; 2.13–2.85)
Influenza B virus	13		
Parainfluenza viruses 1-3	7		
RSV	13		
Enterovirus	7		
Adenovirus	7		
Rhinovirus	7		
Human metapneumovirus	7		
Human bocavirus	7		
**No of individuals**	13	**Reactive 3/13**	**Reactive 2/13**

COVID-19: coronavirus disease; NAT: nucleic acid test; No: number; RSV: respiratory syncytial virus; RT-qPCR: real-time RT-PCR; SARS-CoV-2: severe acute respiratory syndrome coronavirus 2.

^a^ The ratio values (between the extinction of the sample and calibrator) are the signals given by the assays. A ratio < 0.8 is considered negative, ≥ 0.8 and < 1.1 inconclusive and ≥ 1.1 positive.

## Discussion

Our results, albeit based on a small number of samples, showed a higher specificity of SARS-CoV-2 IgG ELISA (91.9%) than SARS-CoV-2 IgA ELISA (73.0%), therefore it is not suggested to use the IgA assay for initial screening. However, our results also indicate that the SARS-CoV-2 IgG ELISA (Euroimmun) with automated analysis and LIS can be used for screening of carefully targeted cohorts. Nevertheless, NAT should remain the method of choice for detection of acute SARS-CoV-2 infection.

In this study, serum samples from one patient with HCoV OC43 infection showed a cross-reaction in the SARS-CoV-2 immunoassays. The total number of HCoV samples was low (n = 7), but results are in line with the data reported by Okba et al. [7] and Amanat et al. [8]. Okba et al. [7] studied the specificity of IgG and IgA SARS-CoV-2 S1 ELISAs (research use only (RUO), Euroimmun) with 203 serum samples including 60 serum samples (taken 2–4 weeks after onset of symptoms) from non-SARS HCoV cases (n = 23 OC43; n = 18 NL63; n = 19 229E). Two HCoV OC43-positive samples cross-reacted in IgG and IgA assays, while HCoVs NL63 and 229E did not. In addition, these serum samples were also tested using a non-commercial Middle East Respiratory Syndrome coronavirus (MERS-CoV), S1 IgG ELISA [11] with similar cross-reactive results. Larger studies, including neutralisation assays, will be needed to further assess seroassay performance.

In our study, samples became IgA positive earlier than IgG, while the specificity of the test was lower for IgA. Considering the relatively slow IgG seroconversion, a second convalescent sample is often needed to obtain reliable test results. In acute phase diagnostics, IgA assays could be useful along with IgG in patients presenting with atypical symptoms, or when RT-qPCR repeatedly remains negative in a suspected case. Nicastri et al. [12] reported a paucisymptomatic case with transient mild conjunctivitis and low fever with fluctuating PCR results. However, IgG response was detected when this case was isolated in the hospital along with a positive PCR result. Haveri et al. [13] reported a patient with mild symptoms seroconverting (IgG) to SARS-CoV-2 in 9 days after onset of symptoms. 

Okba et al. [7] reported that IgG and IgA kinetics varied between patients with different disease severity. In that study, cases with a more severe disease developed an antibody response sooner and in higher concentrations. In our study, there were only 13 patients of 39 who were IgG positive with a median of 12 days (range: 5–20) after onset of symptoms and all 13 were also positive for IgA antibodies. The median for IgA alone (24 patients of 39) was 11 days (range: 5–20). As patient numbers were low, we cannot say if there was any clear pattern similar to the phenomenon observed by Okba et al. in our study, but we noted one case with mild disease severity (ID 5) who was still IgG negative 16 days after onset of symptoms. However, this should be considered when interpreting the serological results for e.g. patients with mild disease.

Seroepidemiological studies will be very useful in informing public health measures in the coming months. In contrast, when attempting to retrospectively establish diagnosis in clinical settings based on serology, it is of critical importance to carefully preselect patients. Otherwise, the positive predictive value will be extremely low, and thus, not meaningful in the current epidemiological situation.
